# A Feature Selection Algorithm to Compute Gene Centric Methylation from Probe Level Methylation Data

**DOI:** 10.1371/journal.pone.0148977

**Published:** 2016-02-12

**Authors:** Brittany Baur, Serdar Bozdag

**Affiliations:** Department of Math, Statistics and Computer Science, Marquette University, Milwaukee, Wisconsin, United States of America; University of Texas at San Antonio, UNITED STATES

## Abstract

DNA methylation is an important epigenetic event that effects gene expression during development and various diseases such as cancer. Understanding the mechanism of action of DNA methylation is important for downstream analysis. In the Illumina Infinium HumanMethylation 450K array, there are tens of probes associated with each gene. Given methylation intensities of all these probes, it is necessary to compute which of these probes are most representative of the gene centric methylation level. In this study, we developed a feature selection algorithm based on sequential forward selection that utilized different classification methods to compute gene centric DNA methylation using probe level DNA methylation data. We compared our algorithm to other feature selection algorithms such as support vector machines with recursive feature elimination, genetic algorithms and ReliefF. We evaluated all methods based on the predictive power of selected probes on their mRNA expression levels and found that a K-Nearest Neighbors classification using the sequential forward selection algorithm performed better than other algorithms based on all metrics. We also observed that transcriptional activities of certain genes were more sensitive to DNA methylation changes than transcriptional activities of other genes. Our algorithm was able to predict the expression of those genes with high accuracy using only DNA methylation data. Our results also showed that those DNA methylation-sensitive genes were enriched in Gene Ontology terms related to the regulation of various biological processes.

## Introduction

Methylation of cytosine nucleotides in DNA (hereafter DNA methylation) is involved in cellular differentiation [1], development [2] and has impact in diseases such as cancer [3]. DNA methylation is typically associated with a decrease in gene expression due to its role in blocking transcription factors from binding [4]. It is also speculated that silencing of a gene could precede DNA methylation [4]. DNA methylation is known to have positive correlation with gene expression, as well, particularly in gene bodies [4]. Several studies integrate DNA methylation with gene expression to unravel the role of DNA methylation in gene regulation [5–8].

DNA methylation has a context-dependent effect on gene expression. For instance, Benet et al. showed that DNA methylation around the transcription start site (TSS) is tightly linked to transcriptional silencing [5]. Varley et al. explored the effects of DNA methylation on gene expression in the context of CpG status and genomic position [6]. They found that the correlation of DNA methylation near the TSS is generally negatively correlated with gene expression and DNA methylation in the gene body is positively or negatively correlated depending on CpG status. Rhee et al. also provided an extensive analysis of the effects of DNA methylation on gene expression in different molecular subtypes of breast cancer [7]. They found that there is more positive correlation of gene expression moving upstream of the TSS in less aggressive subtypes of breast cancer compared to more aggressive subtypes.

A few studies integrate DNA methylation and other data types to predict gene expression. Benet et al. used decision trees to investigate the combinatorial effects of DNA methylation status in different genomic positions on gene expression and found CpG islands to be the most informative feature [5]. Li et al. tested various models to predict gene expression using epigenomics data in lung cancer [9]. They found that a model comprised of 67 features chosen with a ReliefF feature selection and random forest classification performs the best. The set of features is comprised of predominately histone H3 methylation modification and CpG methylation data.

There are several next-generation sequencing-based assays to measure DNA methylation such as bisulfite sequencing, MeDIP-seq, and reduced representation bisulfite sequencing. There are also bisulfite microarray-based assays to measure DNA methylation. For humans, the Illumina Infinium HumanMethylation27 BeadChip Kit array contains 27,578 probes for 14,495 genes [10]. Later, Illumina developed higher-resolution Illumina Infinium HumanMethylation450 BeadChip Kit array, which have an average of 18 probes associated with a gene in various genomic positions and CpG statuses. Due to its high resolution and low cost, the Illumina Infinium HumanMethylation 450K array has become one of the most frequently used assay to quantify DNA methylation in human. At the time of writing the Gene Expression Omnibus database had about 30,000 samples that were profiled using the Illumina 450K array.

Knowing the overall DNA methylation level of a gene is important for downstream functional analysis, such as analyzing regions where DNA methylation blocks transcription factors [7] or determining if a gene has aberrant DNA methylation in cancer [11]. However, it is not straightforward to determine which probes to choose from a 450K array that best represent the overall methylation level of the gene and are informative to the gene’s expression level. A simple, but valuable approach may be to choose a probe based on a metric such as the variation. One approach is to use the standard deviation (SD) across samples and choose the probes with the greatest variation [12, 13]. Other studies restrict the analysis to probes from CpG islands in upstream regions [14], since DNA methylation blocking transcription factors from binding is a well-studied phenomenon. Several studies restrict the number of probes to those within a certain proximity surrounding the TSS [15, 16]. However, this approach ignores possibly informative DNA methylation in the gene body.

In this study, we developed a feature selection algorithm based on sequential forward selection that can utilize various classification methods to select probes that are relevant to gene expression from a 450K array. We also tested this algorithm against more sophisticated approaches such as support vector machines with recursive feature elimination (SVM-RFE), a genetic algorithm and ReliefF. Additionally, we compared our algorithm against several selection methods that do not use gene expression to inform the selection. These methods include choosing the probe with the greatest variation, choosing probes close to the TSS, and choosing probes in upstream CpG islands. Following the selection of probes, we computed a number of metrics to evaluate the prediction quality of gene expression by the selected probes. These metrics included precision, recall, specificity and Matthew’s correlation coefficient. Our results showed that our sequential forward selection algorithm performed best on all metrics when using K-Nearest Neighbors (KNN) where K = 1 (1NN). Our algorithm generally selects one or two probes for each gene, which allows to identify key regions where DNA methylation changes have impact on gene expression.

We also observed that our algorithm could determine genes whose expression levels are putatively sensitive to the changes in their DNA methylation. We showed that these DNA methylation-sensitive genes were enriched for Gene Ontology (GO) terms related to the regulation of various biological processes. Additional functional analysis clustering showed that DNA methylation-sensitive genes also regulated other genes and proteins by a variety of mechanisms, including DNA-binding, kinase activity, protein degradation and protein synthesis.

## Materials and Methods

### Data

Agilent whole genome microarray data and Illumina 450K DNA methylation data of 25 breast cancer lines after treated with the hypomethylating agent, 5-azacitidine (aza) for 72 hours were downloaded from [14] (GSE57343). Log10 Mock/Aza expression data were normalized to account for the different cell lines [14] using LoEss normalization in the LIMMA package [17, 18]. To perform binary prediction of gene expression, the expression data were discretized into up, down and baseline categories using 1.1 fold change threshold for aza-treated cells with respect to mock trials (mock/aza). Baseline mock/aza values were removed. The up and down-expressed mock/aza samples were the binary classifiers in the classification algorithms.

To verify the results of our algorithm on breast cancer cell line, we also downloaded Illumina 450K DNA methylation and Agilent mRNA expression data for 99 Luminal A breast cancer samples from the Cancer Genome Atlas repository [19, S1 File]. Batch effects were corrected in the mRNA expression data using the LIMMA package [18]. Expression data were discretized with a log2 1.2 fold change of the expression level of the sample over the median expression level for that gene across samples. We used the 1.2 fold change threshold instead of 1.1 in tissue samples to reduce potential noise in the discretized data. Baseline sample expression/median expression values were removed. The up and down-expressed sample expression/median expression were the binary classifiers in the classification algorithm.

### A sequential feature selection algorithm for classification methods

We developed a sequential feature selection (SFS) algorithm that can use different classification methods to select the probes that are most relevant to gene expression (Algorithm 1) [20]. SFS sequentially adds features until there is no improvement in the prediction. The objective function of the SFS algorithm is the minimization of the mean classification error in a 10-fold cross-validation (CV).

Algorithm 1 describes the process for a single gene and a set of *n* probes associated with the gene, *X*. Given the DNA methylation levels of the probes, *M*_*k*,*X*_, and the associated gene expression levels, *y*_*k*_, each probe is individually tested in a 10-fold cross validation predicting the gene expression based on the DNA methylation levels of the probe (steps 1–5). In each partition of the 10-fold cross validation, the specified classification algorithm (described below) is trained on the training samples. The expression levels of test samples are predicted based on the trained classification algorithm and the methylation levels of the test samples. The number of test samples in which the predicted expression level does not match the true expression level is *O*. *O* is computed for every partition and the mean(*O)* is the classification error, *CCE*. The probe with the best performance, or minimal CCE, in the 10-fold cross validation is selected (steps 6–8).

Additional probes are sequentially added from the pool of remaining probes if the performance in a 10-fold cross validation improves and more samples are predicted correctly (steps 9–18). If no additional probes lead to increased performance, the algorithm is terminated (steps 19–21).

#### Algorithm 1. Sequential feature selection with 10-fold CV

**Input**: *y*_*k*_: discretized up/down gene expression of sample size *k*

*X = (x_1_, x_2,_ ….x_n_): n* potential probes associated with gene to be added to *S*

*M_k,X_*: DNA methylation values for n probes associated with gene in *k* samples

*S*: current set of selected probes, initially empty

*C*: Classification model based on training folds in 10-fold CV

*C = Classification (M_train,S_, y_train_)*,

*O(M_test,S_, y_test_) = sum(y_test_ ≅ predict(C, M_test,S_))*

*Current classification error (CCE): A vector of classification errors for probes being tested*, *the classification error is mean(O) from a 10-fold CV*

*1*. *For i = 1:n*

*2*.        Select probe *x*_*i*_

3.        Compute 10-fold CV. In each partition, compute *C* on training and *O* on test data

4.        Take mean *O* as current classification error, CCE(i)

*5*. *End*

6. Find *j* s.t. *CCE(j)* < *CCE(i)*, 1 ≤ *i* ≤ *n*, *i* ≠ *j*

7. Move probe *x*_*j*_ from *X* to *S*

8. Old classification error, *OCE = CCE(j)*

*9*. *While (true)*

*10.       For each x*_*i*_ ∈ *X*

*11*.              Select probes {*x*_*i*_} ∪ *S*

12.              Compute 10-fold CV. In each partition, compute *C* on training and *O* on test data.

13.              Take mean *O* as current classification error, *CCE(i)*

14.       *End For*

15.       Find *j* s.t. *CCE(j)* < *CCE(i)*, 1 ≤ *i* ≤ *|X|*, *i* ≠ *j*

*16.       If CCE(j) < OCE*17. Move probe *x*_*j*_ from X to S

18.              *OCE = CCE(j)*

*19.       Else*:

*20*.              Stop search

*21. End While*

We used the following classification algorithms in combination with sequential feature selection (Algorithm 1).

#### Support vector machine (SVM)

A linear kernel function was used to map the training data to the kernel space [21]. Sequential minimal optimization was used to find the separating hyperplane.

#### K-Nearest neighbors (KNN)

KNN classification algorithm was applied with K = 1,3 and 5 (1NN, 3NN and 5NN, respectively). A Euclidean distance metric was used for all instances of KNN [22].

#### Decision trees (DT)

The minimum parent size (number of observations) was 10 and the minimum leaf size was 1 [23].

#### Naïve Bayes (NB)

A kernel distribution was specified for predictors in the Naïve Bayes classification algorithm [24].

We also tested other feature selection algorithms, SVM with recursive feature elimination (SVM-RFE), a genetic algorithm feature selection with KNN classification (GA-KNN) and ReliefF.

#### SVM-RFE

The SVM-RFE algorithm was adapted from [25]. This study used a correlation bias reduction strategy to deal with highly correlated features. In our adaptation, we also included a modification to deal with class imbalances, such that the weight of misclassifying the minority class was higher. The weights of the penalties were obtained by solving the equation *n*0 * *w*0 = *n*1 * *w*1, where n0 and n1 were the number of down and up expressed samples, and *w0* and *w1* were the respective weights. We used a Gaussian kernel and ranked the features. For each gene, we selected the top *k* probes where *k* equals to the number of probes selected in the SFS algorithm.

#### GA-KNN

A genetic algorithm for selecting features was adapted from [26]. The goal of the GA algorithm was to minimize the fitness function: $\frac{resubLoss}{N-S}$, where *resubLoss* is the resubstitution loss in a KNN classification (fraction of misclassified data), *N* is the total number of features and *S* is the number of selected features. The denominator of the equation penalizes a large number of selected probes. We tested the algorithm using *K* = 1, 3 and 5.

#### ReliefF

A KNN-based ReliefF implementation from the MATLAB statistics toolbox [20] was also tested. The nearest “hit” of a feature vector for a sample was defined as the closest sample of the same class by Euclidean distance. The nearest “miss” of a feature vector for a sample was defined as the closest sample of the other class. For each iteration, a vector of features from a random instance is selected. The weight of the feature *i* is updated according to the function:

$$W_{i}=W_{i}-{\left(x_{i}-h_{i}\right)}^{2}+{\left(x_{i}-m_{i}\right)}^{2}$$

where *x*_*i*_ is the value of the feature of the randomly selected instance, *h*_*i*_ is the nearest hit and *m*_*i*_ is the nearest miss. Therefore, the weight of a feature decreases if it is more distant from nearby instances of the hits relative to the misses.

We tested this algorithm with K = 1, 3 and 5. This implementation ranks the predictors in order of importance. For each gene, we selected the top *k* probes where *k* equals to the number of probes selected in the SFS algorithm.

We also developed two control algorithms namely random and top two correlated.

#### Random

For a given gene, we randomly selected probes associated with the gene. We set the number of probes randomly selected for a gene equal to the number of probes that were selected in the SFS algorithm that we compared to.

#### Top two correlated

The two probes most positively or negatively correlated with gene expression were selected.

We tested our algorithm against following probe selection methods, which do not take into account gene expression.

#### All

For a given gene, all the probes associated with the gene are selected.

#### Upstream CpG Island

For a given gene, we selected probes that are in CpG islands in the upstream regions (TSS200, TSS1500, 5’ UTR and 1^st^ Exon).

#### TSS

For a given gene, we selected probes within a 2500bp window of the transcription start site.

#### Top SD

For a given gene, the probe with the highest standard deviation is selected.

### Assessment of algorithms

We calculated various metrics to test each algorithm’s ability to predict gene expression based on the selected DNA methylation probes. We applied a leave-one-out cross validation (LOO-CV) with an appropriate model using the selected probes as predictors and the discretized gene expression as a response. For the SFS algorithm, the classification model used in the feature selection was used in the LOO-CV. For GA-KNN and ReliefF, KNN was used in the LOO-CV. For SVM-RFE, SVM was used in the LOO-CV. For the methods that do not integrate gene expression, namely All, Upstream CpG Island, TSS and Top SD, we evaluated the probe selection with a LOO-CV using KNN, DT, SVM and NB.

Following the LOO-CV, we computed the number of true positive (TP), true negatives (TN), false positives (FP) and false negatives (FN) and calculated various metrics. We considered down-expressed cases positive and up-expressed cases negative outcomes. We calculated the prediction accuracy ((TP + TN)/(TP+TN+FP+FN)), recall (TP/(TP+FN)), precision (TP/(TP+FP)) and specificity (TN/(TN+FP)) for each method. We also computed Matthew’s correlation coefficient (MCC) [Eq 1]. MCC can be considered a balanced measure of accuracy even when the class sizes may be different.

$$\frac{TPxTN-FP\;x\;FN}{\sqrt{\left(TP+FP\right)\left(TP+FN\right)\left(TN+FP\right)\left(TN+FN\right)}}$$ (1)

### Gene Ontology and functional enrichment

To perform functional analysis on genes whose expression were predicted with high accuracy by DNA methylation, we selected genes that have an MCC > 0.6 in the SFS algorithm. We performed a GO-term enrichment analysis using the web tool GOrilla [27], by comparing the list of genes with high MCC to a background of the full list of 17,043 genes in the dataset. To show that the enrichment of GO terms obtained is specific to genes with high MCC, we compared the list of GO terms and p-values for genes with high MCC to the list of GO terms and p-values for genes with MCC < 0.2.

In order to investigate if there are any functional differences between genes that have gene body and upstream methylation, we performed gene functional classification clustering using DAVID [28]. Given an input gene list, the DAVID’s functional clustering tool generates a gene-to-gene similarity matrix based on shared functional annotations from different sources [29]. A clustering algorithm classifies the genes into functionally related clusters. Each functional cluster contains certain related terms shared between the genes in the group. We separated all genes with MCC > 0.6 based on whether the selected probes by the SFS algorithm were exclusively from upstream regions (gene had probes only in 5’ UTR, 1^st^ Exon, TSS200 or TSS1500 as defined by Illumina) or exclusively from the gene body applied functional clustering using DAVID for each group of genes.

### Implementation

Our algorithm is unbiased as it does not restrict analysis by CpG status or genomic position. We implemented the tool in MATLAB [20]. The source code is freely available under the MIT Open Source license (https://github.com/brittanybaur/genecentricmethylation).

## Results and Discussion

### KNN-SFS algorithm resulted in higher recall and specificity

**Fig 1:**
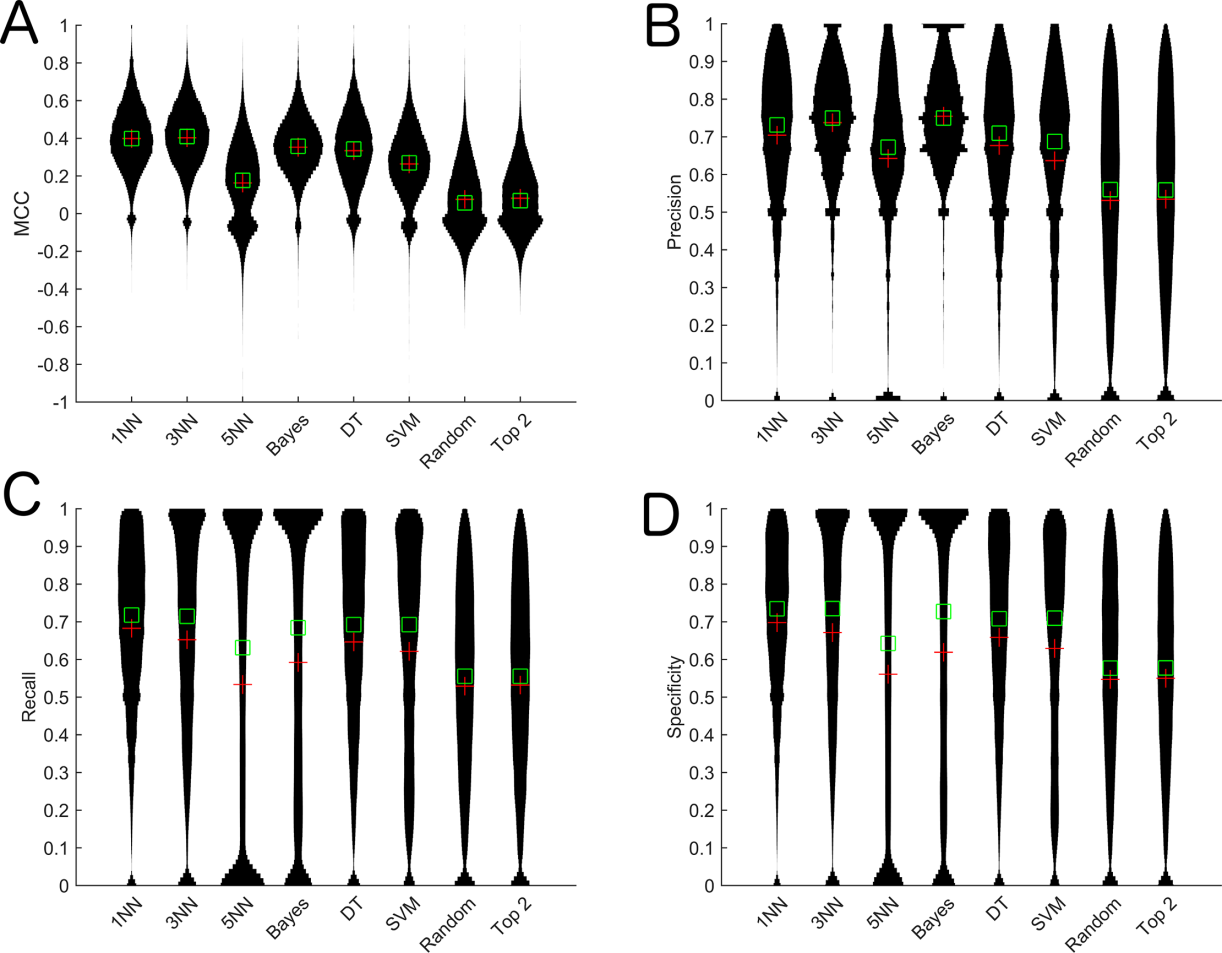
Violin plots of performance metrics for the algorithm when utilizing different classification methods in the SFS algorithm and controls on the breast cancer cell line data. A) MCC, B) Precision, C) Recall, D) Specificity. Green squares specify the median and the red pluses specify the mean. NB: Naive Bayes, DT: Decision tree, SVM: Support Vector Machine

**Fig 2:**
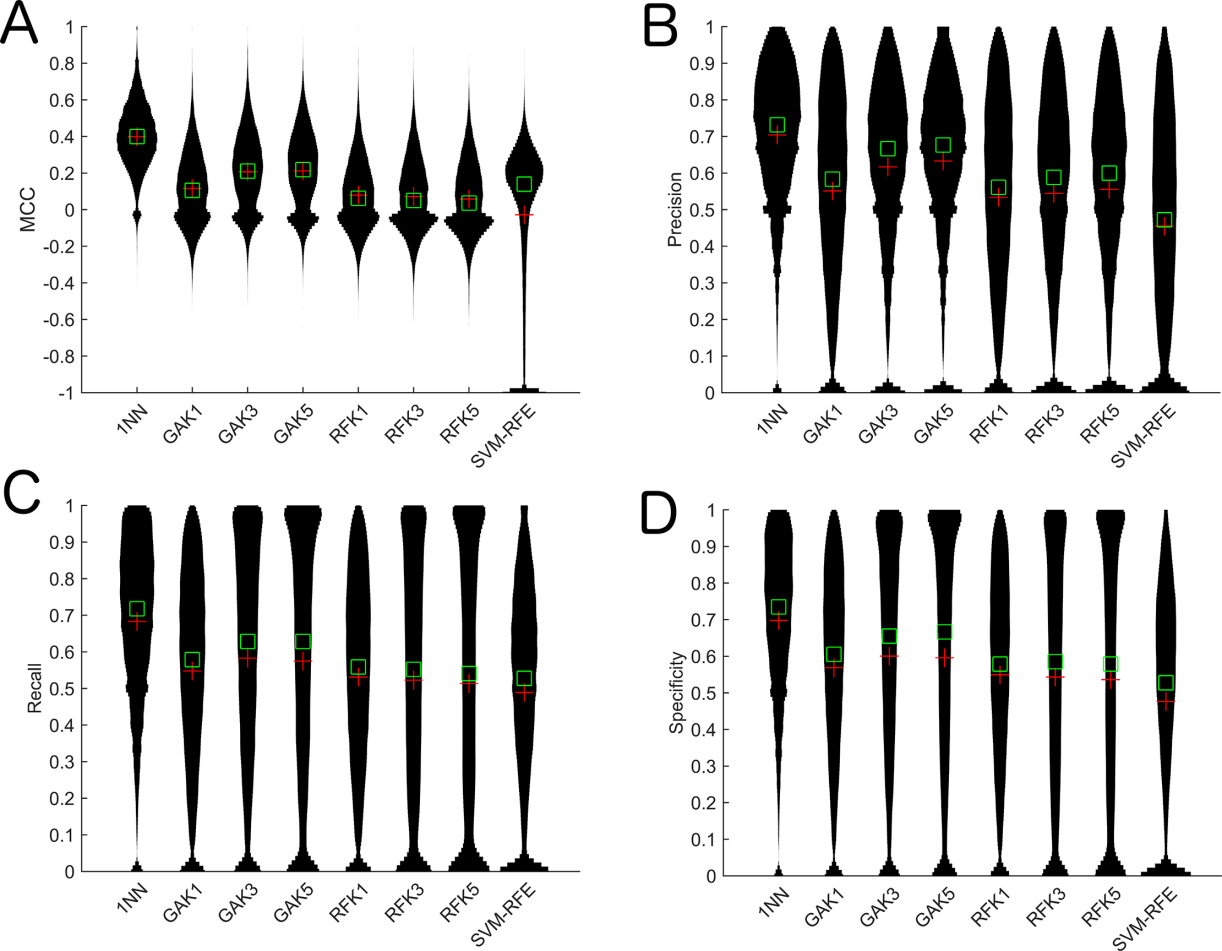
Violin plots of performance metrics for 1NN-SFS algorithm against other algorithms on the breast cancer cell line data. A) MCC, B) Precision, C) Recall, D) Specificity. Random: KNN random, Top 2: KNN top two (see Methods). GAK: GA-KNN algorithm with varying K-nearest neighbors. RFK: Relief-F algorithm with varying K nearest neighbors.

We calculated the prediction accuracy, specificity, recall, precision and MCC for the SFS algorithm using the four different classification algorithms on 31,171 transcripts on the breast cancer cell line data obtained from [14]. We calculated various metrics such as precision, recall, specificity and MCC due to the class imbalance of up/down expressed samples (S1 Fig). We found that the 1NN-SFS algorithm resulted in the highest MCC, recall and specificity, and the third highest precision (Table 1, Fig 1). The 1NN algorithm also resulted in the second highest accuracy (S2A Fig). We compared the 1NN-SFS algorithm to the random and top two correlated selection methods and evaluated the predictive performance of the probe selection with a 1NN-based LOO-CV. To ensure a fair comparison, we set the number of probes selected for a gene in the 1NN-Random method equal to the number of probes selected for that gene in the 1NN-SFS algorithm. We found that all these controls resulted in worse performance than our algorithm. We also compared 1NN-SFS algorithm to GA-KNN and ReliefF algorithms for K = 1, 3 and 5, and to the SVM-RFE algorithm. We set the number of top ranked probes selected in ReliefF and SVM-RFE equal to the number of probes selected by 1NN-SFS. We observed that the 1NN-SFS algorithm performed better than GA-KNN and ReliefF algorithms for K = 1, 3 and5, and the SVM-RFE algorithm by all metrics (Fig 2, S2B Fig). Taken together, these results indicate that the 1NN-SFS feature selection method chooses more relevant probes than other algorithms.

**Table 1:** Mean performance of SFS algorithms and controls on the breast cancer cell line data.

	1NN	3NN	5NN	NB	DT	SVM	1NN Random	1NN Top Two
Accuracy	0.79	0.80	0.74	0.78	0.77	0.74	0.66	0.67
Precision	0.70	0.74	0.65	0.75	0.68	0.64	0.53	0.54
Recall	0.68	0.65	0.53	0.59	0.65	0.63	0.53	0.53
Specificity	0.70	0.67	0.56	0.62	0.66	0.63	0.55	0.55
MCC	0.40	0.40	0.16	0.35	0.33	0.26	0.08	0.08

**Fig 3:**
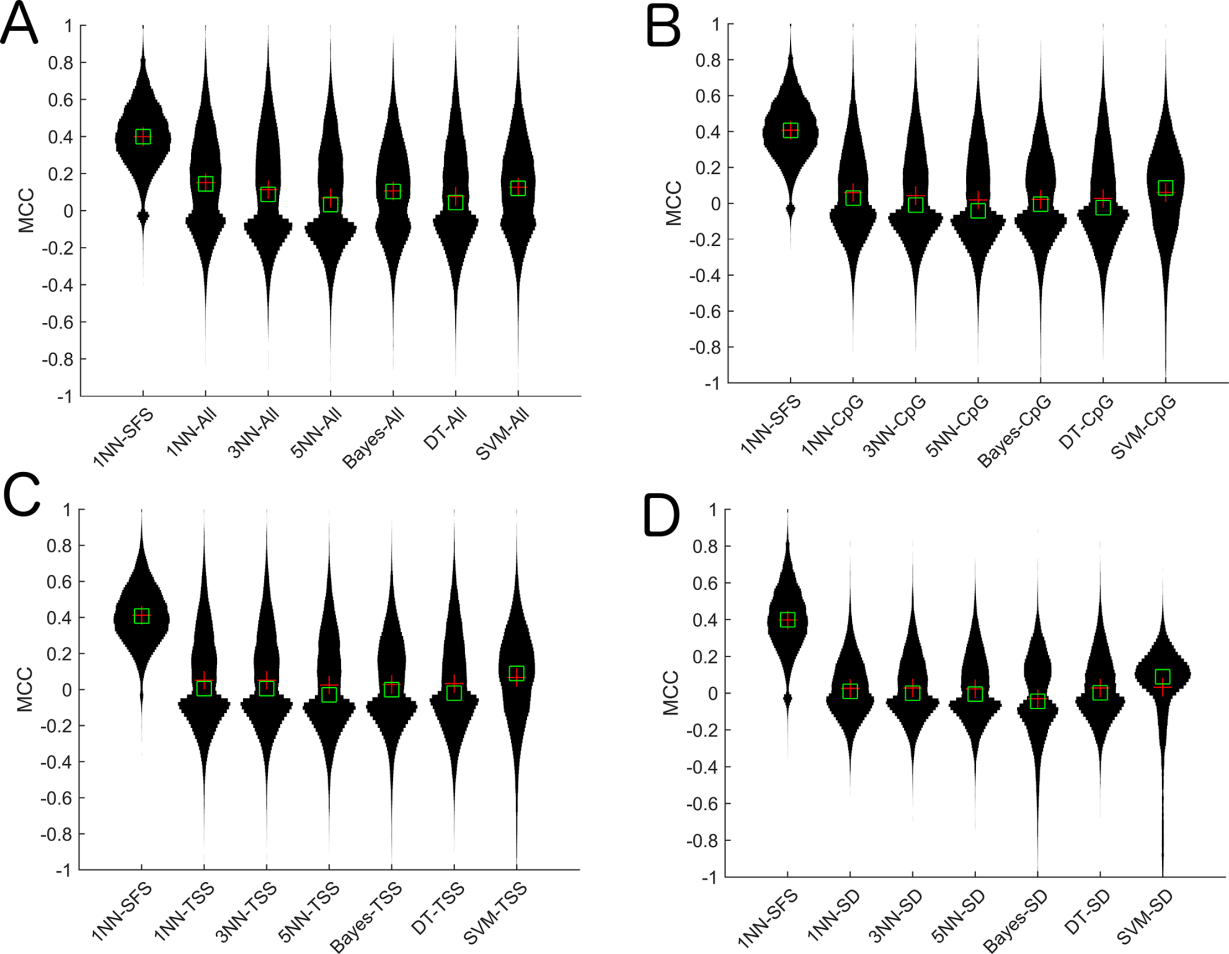
Violin plots of MCC for 1NN-SFS algorithm against other probe selection methods on the breast cancer cell line data. A) All, B) Upstream CpG Island, C) TSS, D) Top SD.

We compared the 1NN-SFS algorithm to probe selection methods that do not take into account gene expression. All of these approaches to probe selection resulted in significantly lower performance when compared to the 1NN-SFS algorithm that integrate gene expression, suggesting the importance of integrating gene expression data to inform the probe selection (Fig 3).

**Fig 4:**
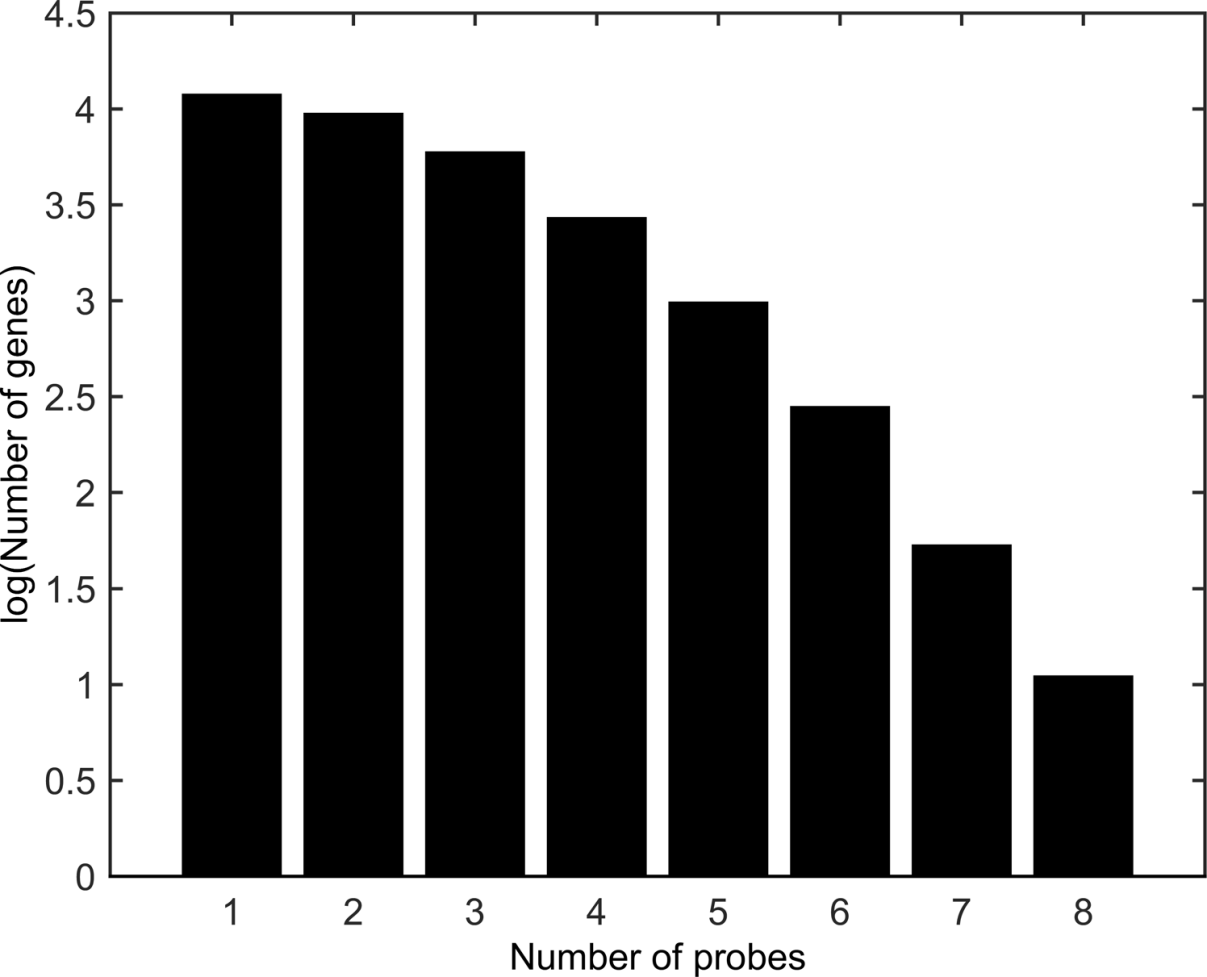
Number of probes selected per gene by 1NN-SFS algorithm on the breast cancer cell line data.

We observed the 1NN algorithm usually only selected one or two probes per gene (Fig 4). Out of the 31,171 transcripts tested, 11,833 transcripts had one probe selected and an additional 9,411 transcripts had two probes selected. Since selecting all of the probes leads to significantly poorer performance, the selection of the best one or two probes is important to the algorithm’s good performance. This shows that our algorithm was able to reduce the number of probes for a given gene to a limited number of key informative probes.

### KNN algorithm resulted in consistent prediction accuracy

**Fig 5:**
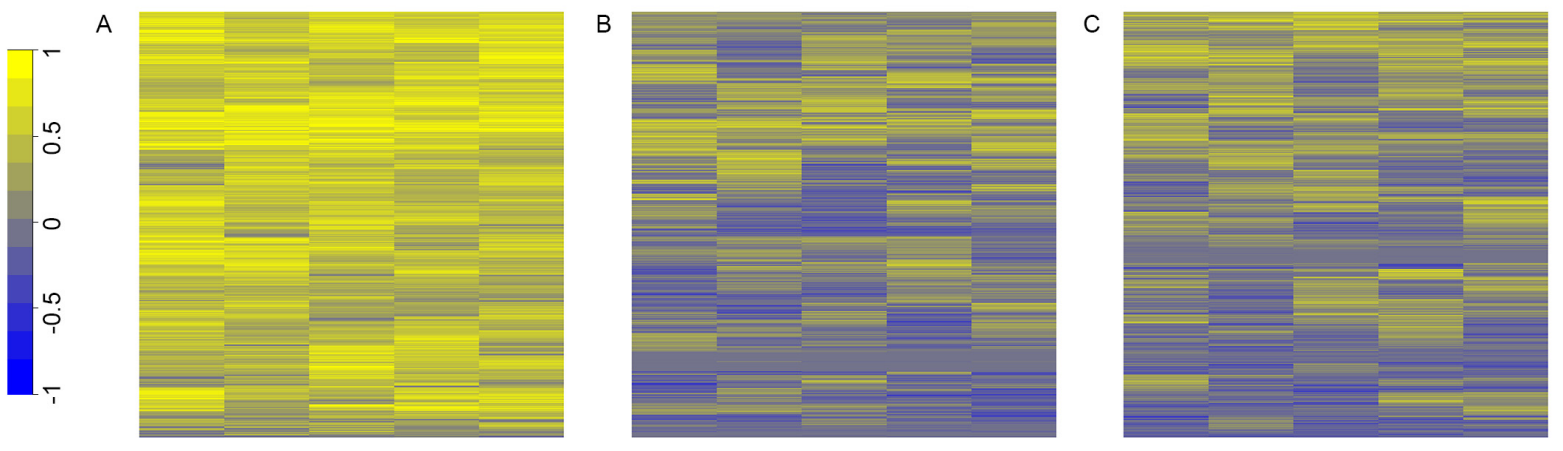
Heatmap clustering of MCC values. Heatmap clustering of MCC values for five executions of the algorithm on random halves of the breast cancer cell line data for A. 1NN algorithm and B. random selection of probes C. Top two correlated approach.

To check the consistency of the algorithm on smaller subsets of the data, we ran the algorithm five additional times on half of the dataset, in which the samples were randomly chosen each execution. For each of the five executions, we compared 1NN-SFS algorithm to random selection method and top two correlated method. Fig 5 shows a heatmap comparison of the MCC for the five runs of the 1NN algorithm compared to the random selection and top two correlated selection. The 1NN consistently gave higher MCC values over the random selection and top two correlated selection. Additionally, the MCC values were consistent across runs.

### DNA methylation-sensitive genes were enriched for regulation-based GO terms

We investigated if there are any common functional property on genes whose transcription levels are sensitive to DNA methylation changes by analyzing genes where the selected probes predict gene expression well. 3,084 genes had MCC > 0.6 in the 1NN-SFS algorithm. The GOrilla results are summarized in Table 2. Table 2 shows that DNA methylation-sensitive genes were enriched in GO terms related to the regulation of various biological processes. The table only encompasses only the top 30 significant GO terms.

**Table 2:** Top 30 GO Terms for genes with MCC >0.6 by 1NN-SFS algorithm on the breast cancer cell line data.

Description	FDR q-value
regulation of multicellular organismal process	4.43E-19
regulation of developmental process	2.51E-17
regulation of multicellular organismal development	9.31E-17
positive regulation of biological process	1.16E-16
movement of cell or subcellular component	1.23E-16
positive regulation of cellular process	1.41E-16
negative regulation of biological process	2.3E-16
anatomical structure development	1.38E-15
negative regulation of cellular process	2.72E-15
regulation of cell differentiation	2.85E-15
cell migration	6.81E-15
negative regulation of metabolic process	2.48E-14
anatomical structure morphogenesis	3.53E-14
organ development	5.3E-14
transmembrane receptor protein tyrosine kinase signaling pathway	6.02E-14
cell motility	7.21E-14
Locomotion	1.7E-13
developmental process	1.71E-13
enzyme linked receptor protein signaling pathway	1.75E-13
single-organism developmental process	1.76E-13
regulation of cell development	2.88E-13
regulation of anatomical structure morphogenesis	4.5E-13
negative regulation of macromolecule metabolic process	6.04E-13
intracellular signal transduction	8.58E-13
single-multicellular organism process	2.36E-12
multicellular organismal process	5.86E-12
regulation of localization	1.06E-11
positive regulation of multicellular organismal process	1.07E-11
signal transduction	1.27E-11
cellular component organization or biogenesis	1.39E-11
positive regulation of developmental process	3.2E-11

To verify that this result is specific to well-predicted genes, we compared the result to poorly-predicted genes. We performed GO analysis on 2,880 genes that have MCC < 0.2. We chose MCC thresholds carefully to ensure a fair comparison to GO analysis by having comparable gene set sizes. Table 3 shows that only immune response and stimulus detection terms are reported as significant. This result suggest that enrichment of regulation-related GO terms are specific to genes with high MCC values.

**Table 3:** GO terms with MCC < 0.2 for genes by 1NN-SFS algorithm on the breast cancer cell line data.

Description	FDR q-value
detection of chemical stimulus involved in sensory perception of smell	5.62E-11
detection of chemical stimulus involved in sensory perception	5.74E-11
detection of chemical stimulus	5.62E-8
detection of stimulus involved in sensory perception	1.07E-7
detection of stimulus	1.95E-3
immune response	1.23E-2

We applied DAVID’s functional classification tool on genes with MCC > 0.6 to determine functional enrichment differences for genes with selected gene body probes and genes with selected promoter probes. 1035 genes had exclusively upstream probes selected, resulting in 33 functional clusters. 699 genes had exclusively gene body probes selected, resulting in 27 functional clusters. We found that in both the promoter and gene body group, many of the clusters suggested that the genes are involved in the regulation of other genes and proteins via a variety of mechanisms. The most enriched clusters are summarized in Tables 4 and 5. S1 Table and S2 Table contains a full list of the top clusters.

**Table 4:** Functional clusters of genes with MCC > 0.6 with upstream probes selected by 1NN-SFS algorithm on the breast cancer cell line data.

Cluster Number	Number of genes	Enrichment	Most significant terms (p-val)	Other representative terms (p-val) and notes
1	40	4.39	Atp-binding (4.4E-45), Nucleotide-binding (4.6E-38), adenyl ribonucleotide binding (1.7E-37)	Helicase (4E-12), kinase (5.8E-6), protein kinase activity (3.7E-4)
2	4	3.67	Repeat:ANK 1 (1.7E-6), Repeat:ANK 2 (1.8E-6), Ankyrin (2.9E-6)	Genes coding for ankyrin proteins
3	45	3.46	Kinase (1.8E-56), Protein Kinase–ATP binding site (2.0E-56), domain: protein kinase (2.1E-53)	Phosphorylation (1.7E-51), transferase (1.1E-47), nucleotide binding (2.1E-34)
4	13	3.42	Microtubule cytoskeleton (9.6E-15), cytoskeleton (9.1E-14), cytoskeletal part (4.1E-12)	Centrosome (2.3E-8), genes involved in regulation of cell motility
7	4	2.91	binding site:S-adenosyl-L-methionine (1.8E-8), s-adenosyl-l-methionine (1.5E-7), methyltransferase (4.3E-7)	Genes coding for methyltransferases
8	5	2.83	Microfilament motor activity (22.0E-12), actin filament-based movement (6.3E-12), domain:Myosin head-like (9.4E-12)	Genes coding for myosin proteins
9	6	2.66	Anti-apoptosis (7.8E-12), negative reglation of apoptosis (1.2E-8), negative regulation of programmed cell death (1.3E-8)	Genes predominately related to BCL2 (BAG3, BAG4, BCL2A1, BL210). Also includes MCL1 and TNFRSF10D
10	16	2.54	Nucleotide phosphate-binding region:GTP (4.7E-28), gtp-binding (2.3E-27), Ras (2.7E-16)	Genes predominately related to the RAS oncogene family
13	59	2.29	Transcription regulator activity (2.7E-50), transcription regulation (2.2E-47), regulation of transcription, DNA dependent (2.2E-47)	Sequence specific DNA-binding (3.1E-29), repressor (6.0E-22)
19	10	1.72	Ribosomal protein (6.7E-19), structural constituent of ribosome (8.2E-18), cytostolic ribosome (1.6E-17)	Genes coding for ribosomal proteins

**Table 5:** Functional clusters of genes with MCC > 0.6 with gene body probes selected by 1NN-SFS algorithm on the breast cancer cell line data.

Cluster Number	Number of genes	Enrichment	Most significant terms (p-val)	Other representative terms (p-val) and notes
1	48	2.84	Atp-binding (1.1E-51), Nucleotide-binding (6.5E-47), adenyl ribonucleotide binding (4.2E-45)	phosphorylation (4.8E-33), kinase (7.6E-40), transferase(1.9E-29)
2	12	2.36	Nucleolus (1.2E-14), nuclear lumen (3.9E-11), intracellular organelle lumen (3.7E-10)	
3	11	2.06	Transcription regulation (1.6E-10), transcription(2.1E-10), regulation of transcription (6.8E-8)	
4	9	1.83	Ribosomal protein (7.2E-17), ribonucleoprotein (1.8E-15), ribosome (5.6E-15)	RNA binding (2.8E-4)
8	6	1.39	Negative regulation of ubiquitin-protein ligase activity during mitotic cell cycles (2.2E-12), negative regulation of ubiquitin-ligaase activity (2.6E-12)	Genes coding for proteasomes and ubiquitin
9	66	1.38	Regulation of transcription (1.1E-34), transcription (2.4E-24), transcription regulation (5.0E-32)	

For genes with probes selected from the promoter regions (Table 4), the most enriched cluster contains genes involved in ATP-binding, nucleotide-binding, helicase and protein kinase activity. Additionally, cluster 3 also contains many kinase, phosphorylation and nucleotide binding terms. A common theme is that these terms are all mechanisms by which other genes and proteins can be regulated. Importantly, these functions may be related to the regulation-based GO terms represented in the GOrilla analysis. Other possible mechanisms of regulation of other genes and proteins include an enrichment of DNA-methyltransferases (cluster 7) and regulation of protein synthesis via ribosomal protein (cluster 19). DNA methylation may also play a role in the regulation of apoptosis-related genes (cluster 9) and cell motility (cluster 8). A group of 59 genes were enriched in terms related to transcription regulator activity (cluster 13).

Similar results were obtained for genes where the probes were selected from gene body regions (Table 5). The first and third cluster involve transcription regulation and protein kinase activity. Cluster 4 contains additional genes coding for ribosomal proteins. Cluster 8 contains genes coding for proteasomes and ubiquitin, suggesting that protein degradation may also be under the control of DNA methylation of certain genes. Additionally, 66 genes were enriched in terms related to transcription regulation (cluster 9).

Together, these results suggest that if DNA methylation is a good predictor of gene expression (MCC > 0.6) than that gene may likely be involved in the regulation of other genes and proteins through a variety of mechanisms including DNA binding, protein kinase activity, protein synthesis and protein degradation. We did not find a significant functional difference between genes where gene body probes are selected and genes where upstream probes are selected. This suggests that a gene under strong epigenetic control via DNA methylation is more likely to be a regulatory gene, regardless of the genomic position of the predictive DNA methylation.

### Verification in TCGA luminal A breast cancer data

**Fig 6:**
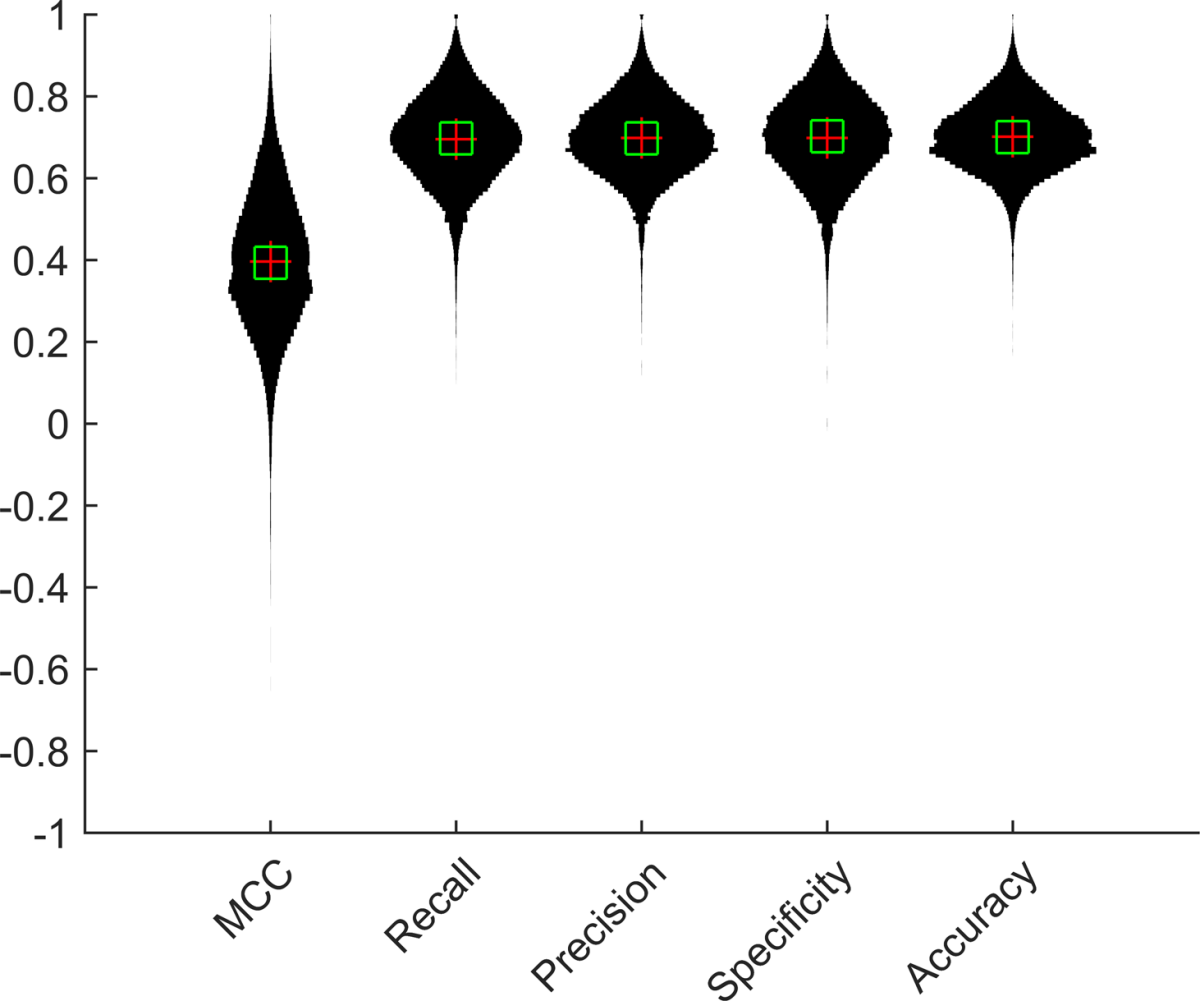
Performance metrics of 1NN-SFS algorithm on TCGA data.

To verify our work in another dataset, we performed the 1NN-SFS algorithm on 99 luminal A breast cancer samples from the TCGA database. We computed the performance metrics, and found the average to be 0.7 for all metrics (Fig 6).

We performed the same GO-term analysis for luminal A data that we performed in the cell line data. We chose 1,823 and 1,407 genes that were predicted with an MCC > 0.6 and MCC < 0.2, respectively. 534 of the genes with MCC > 0.6 in the TCGA data overlapped with the genes with MCC > 0.6 in the cell line data (hypergeometric p-value < 2.01 e-41). Table 6 shows only the top 30 GO terms for genes with high MCC and Table 7 shows all of the GO terms for genes with low MCC. Similar to our previous result for the cell line data, we found that genes that predicted well were again enriched in GO-terms related to the regulation of various biological processes while genes that were predicted poorly were not. We note here that the poorly-predicted genes had GO-terms involved in the detection of a chemical stimulus and smell. This was due to a single family (olfactory receptor family) where almost all of the members of the family had their expression predicted poorly. This was not the case for the regulation-based terms in the well-predicting gene set.

**Table 6:** Top 30 GO terms with MCC > 0.6 for genes by 1NN-SFS algorithm on TCGA data.

Description	FDR q-value
positive regulation of cellular process	3.75E-8
positive regulation of biological process	2E-7
RNA metabolic process	3.6E-7
regulation of metabolic process	7.55E-7
regulation of transcription from RNA polymerase II promoter	8.6E-7
cellular macromolecule metabolic process	9.69E-7
regulation of gene expression	1.16E-6
regulation of macromolecule metabolic process	1.19E-6
regulation of macromolecule biosynthetic process	1.35E-6
regulation of cellular macromolecule biosynthetic process	1.36E-6
RNA biosynthetic process	1.45E-6
regulation of primary metabolic process	1.54E-6
regulation of biosynthetic process	1.56E-6
macromolecule metabolic process	2.36E-6
aromatic compound biosynthetic process	2.48E-6
regulation of cellular biosynthetic process	2.52E-6
positive regulation of RNA biosynthetic process	3.02E-6
regulation of RNA biosynthetic process	3.12E-6
nucleobase-containing compound biosynthetic process	3.4E-6
nucleic acid metabolic process	3.44E-6
regulation of cellular metabolic process	3.45E-6
regulation of transcription, DNA-templated	3.63E-6
cellular process	3.73E-6
heterocycle biosynthetic process	3.93E-6
cellular nitrogen compound biosynthetic process	4.29E-6
positive regulation of macromolecule biosynthetic process	4.35E-6
regulation of nucleic acid-templated transcription	5.11E-6
nucleobase-containing compound metabolic process	6.76E-6
regulation of nucleobase-containing compound metabolic process	1.04E-5
positive regulation of RNA metabolic process	1.07E-5

**Table 7:** GO terms with MCC < 0.2 for genes by 1NN-SFS algorithm on TCGA data.

Description	FDR q-value
detection of chemical stimulus involved in sensory perception	1.27E-42
detection of chemical stimulus	6.29E-41
detection of chemical stimulus involved in sensory perception of smell	8.16E-41
detection of stimulus involved in sensory perception	3.18E-38
detection of stimulus	7.93E-31
G-protein coupled receptor signaling pathway	1.44E-21
sensory perception of smell	1.16E-19
sensory perception of chemical stimulus	4.86E-14
cell surface receptor signaling pathway	7.68E-7
sensory perception	7.02E-6
response to stimulus	5.47E-5
drug metabolic process	4.77E-3
signal transduction	1.12E-2

We performed DAVID’s functional classification analysis on genes with probes exclusively selected from the promoter and genes with probes exclusively selected from the gene body as previously described. 659 genes with MCC > 0.6 contained selected probes exclusively from the upstream regions,resulting in 22 total clusters. 396 genes with MCC > 0.6 contained selected probes exclusively from the gene body, resulting 23 clusters. The results are summarized in Tables 8 and 9. A full list of top enriched clusters are contained in S3 Table and S4 Table. For genes with selected probes from the promoter (Table 8), cluster 2 contained genes involved with RNA splicing, which is another mechanism by which other genes can be regulated. Similar to functional clustering results on cell line data, cluster 4 contained genes coding ribosomal proteins and cluster 1 and 5 contained transcriptional regulation genes. For genes with probes selected from the gene body (Table 9), clusters 1 and 3 had terms involved with protein regulation and cluster 2 contained genes involved with nucleotide-binding. For both the cell line and TCGA data for genes with selected gene body probes, chromodomain helicase and GTP-binding clusters were observed (S2 Table and S4 Table).

**Table 8:** Functional clusters of genes with MCC > 0.6 with upstream probes selected by 1NN-SFS algorithm in TCGA data.

Cluster number	Number of genes	Enrichment	Top terms (pval)	Other representative terms and notes
1	5	4.73	Nucleolus (8.8E-6), nuclear lumen (1.6E-4), intracellular organelle lumen (3.7E-4)	Transcription, DNA-dependent (4.3E-2)
2	24	4.08	RNA splicing (1.0E-29), RNA processing (8.0E-29), mRNA processing (1.1E-28)	Spliceosome (6.8E-23), rna-binding (2.3E-10)
3	13	2.48	Cytoskeleton (1.5E-18), cytoplasm (7.2E-10), microtubule cytoskeleton (4.7E-9)	
4	11	2.25	Ribosomal protein (6.3E-21), ribonucleoprotein (3.5E-19), ribosome (1.5E-18)	Group of genes coding for mitochondrial ribosomal proteins
5	134	2.2	Transcription regulation (1.9E-45), zinc (4.1E-45), transcription (1.3E-43)	Transcription regulation
6	13	2.03	Ubl conjugation pathway (1E-19), modification-dependent protein catabolic process (3E-17), modification-dependent macromolecule catabolic process (3E-17)	Ubiquitin proteins, proteolysis (4.7E-14)

**Table 9:** Functional clusters of genes with MCC > 0.6 with gene body probes selected by 1NN-SFS algorithm in TCGA data.

Cluster Number	Number of genes	Enrichment	Most significant terms (p-val)	Other representative terms (p-val) and notes
1	4	3.3	GTPase activation (5.5E-7), domain:PH (1.9E-6), Pleckstrin homology (4.5E-6)	Rho GTPases
2	5	2.5	Atp-binding (2.2E-5), nucleotide-binding(5.9E-5), adenyl ribonucleotide binding (1.8E-4)	
3	17	2.14	Protein kinase–core (8.7E-23), kinase (2.7E-21), protein kinase–atp binding site (1.2E-20)	Phosphorylation (1.9E-20), nucleotide-binding (1.9E-15), transferase (7.3E-16)
4	5	1.86	Zinc (1.7E-4), metal-binding (5.7E-4), zinc ion binding (1E-3)	

## Conclusions

We developed an algorithm, which utilizes different classification and regression methods to select DNA methylation probes from the Illumina Infinium HumanMethylation450 BeadChip Kit array that are most relevant to expression of their corresponding gene. We tested the algorithms based on their ability to predict up/down expressed samples. We found that the 1NN-SFS algorithm performed the best compared to other methods tested (Figs 1 and 2) and random selection (Fig 1). We demonstrated that this algorithm led to consistent results (Fig 5). The 1NN-SFS has the advantages of selecting a certain number of probes as opposed to ranking the probes.

We also observed that genes whose expression was predicted by DNA methylation with high accuracy were enriched in GO terms related to the regulation of various biological processes in both datasets. The overlap between highly predicted genes in both datasets were also significantly higher. Genes whose expression was accurately predicted by DNA methylation may be more sensitive to changes in DNA methylation. Therefore, genes that are sensitive to changes in DNA methylation may be more likely to be involved in the regulation of various biological processes.

Additionally, functional clustering revealed that many genes that were sensitive to DNA methylation were regulators of other genes and proteins through a variety of mechanisms including DNA-binding, protein kinase activity, protein degradation and protein synthesis. These results suggest that these functions may answer how genes under the control of DNA methylation regulate the various biological processes. There were no significant differences in function between genes with gene body probes selected and genes with upstream probes selected. This suggests that genes under the control of DNA methylation are more likely to be a regulatory gene regardless of the genomic position of the most predictive DNA methylation.

In order to verify results on cell line dataset, we also applied 1NN-SFS on a breast cancer dataset obtained from TCGA. The overall prediction accuracy in breast cancer data was lower than the accuracy in cell line data (Figs 1 and 6). This could be due to the heterogeneity of the tissue samples. The expression of the tissue samples might be affected by other factors such as copy number alteration and mixed cell population in the tissues. On the other hand, cell line data contain more homogenous cells in each sample.

These methods will help researchers evaluate which probes are most involved in gene expression and which genes are sensitive to changes in DNA methylation. Future work should be aimed at studying other DNA methylation platforms to find the best methods for choosing regions of where DNA methylation has a significant impact on gene expression. The ideas in this paper could be extended to bisulfite sequencing and other commonly used platforms. Methylation-seq data could work if the data is converted to segment data. Additionally, the combinatorial effects of DNA methylation in different regions on gene expression can be studied with approaches similar to methods here.

## Supporting Information

S1 Fig Heatmap of discretized mock/aza expression data in breast cancer cell lines. Red: Up-expressed, Green: Down-expressed, Black: Baseline.
(TIF)

S2 Fig Accuracy of probe selection methods that integrate gene expression data. (TIF)

S3 Fig Heatmap of discretized expression data in TCGA Luminal A breast cancer datasets. Red: Up-expressed, Green: Down-expressed, Black: Baseline.
(TIF)

S1 File TCGA IDs for 99 Luminal A samples used in the analysis. (TXT)

S1 Table Top 20 functional clusters of genes with MCC > 0.6 with upstream probes selected. (DOCX)

S2 Table Functional clusters of genes with MCC > 0.6 with gene body probes selected with enrichment > 1. (DOCX)

S3 Table Functional clusters of genes with MCC > 0.6 with upstream probes selected in TCGA data with enrichment > 1. (DOCX)

S4 Table Functional clusters of genes with MCC > 0.6 with gene body probes selected in TCGA data with enrichment > 1. (DOCX)
