# Supplementary figures and images for: A Feature Selection Algorithm to Compute Gene Centric Methylation from Probe Level Methylation Data
Source: PLoS One. 2016 Feb 12;11(2):e0148977. doi: 10.1371/journal.pone.0148977 (PMC4752315; doi:10.1371/journal.pone.0148977)

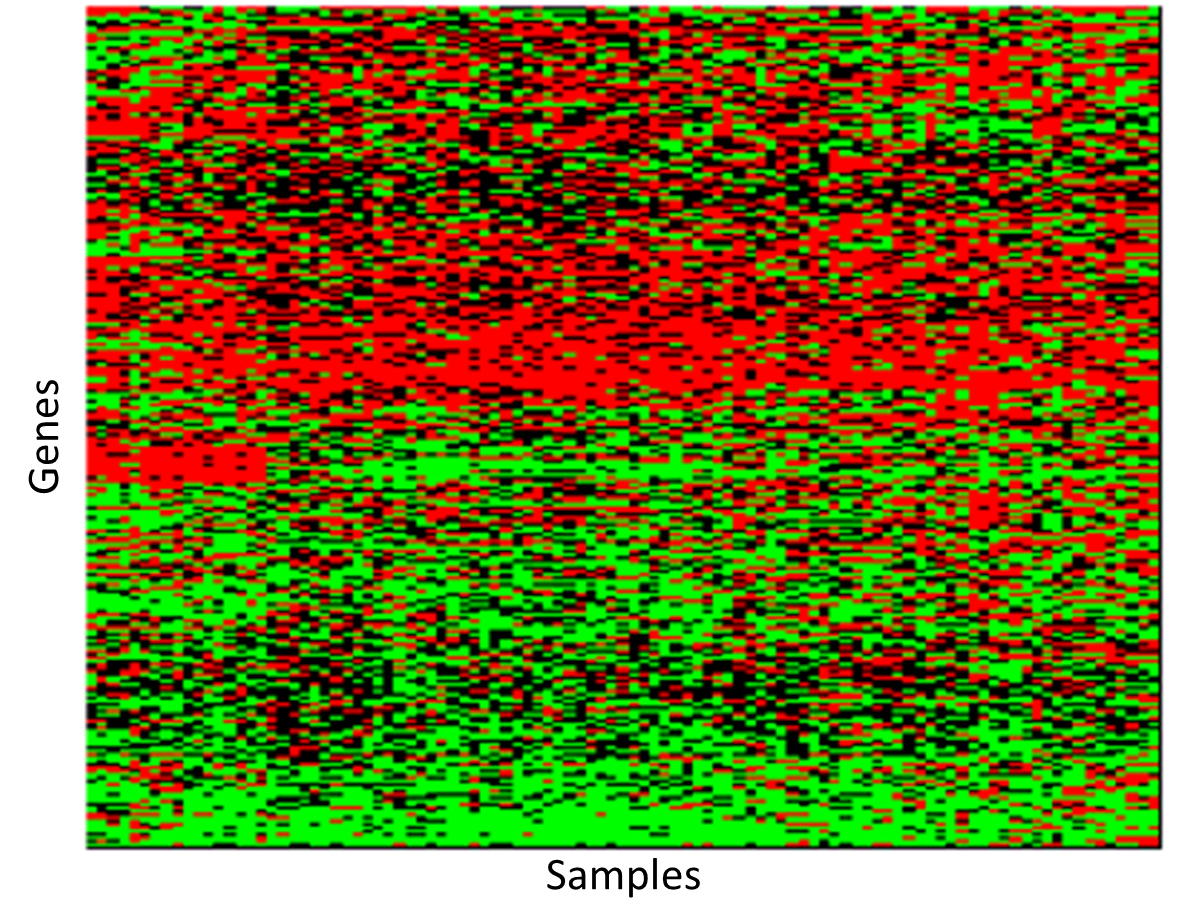

Supplement: S1 Fig — Red: Up-expressed, Green: Down-expressed, Black: Baseline. (TIF) [file pone.0148977.s001.tif]

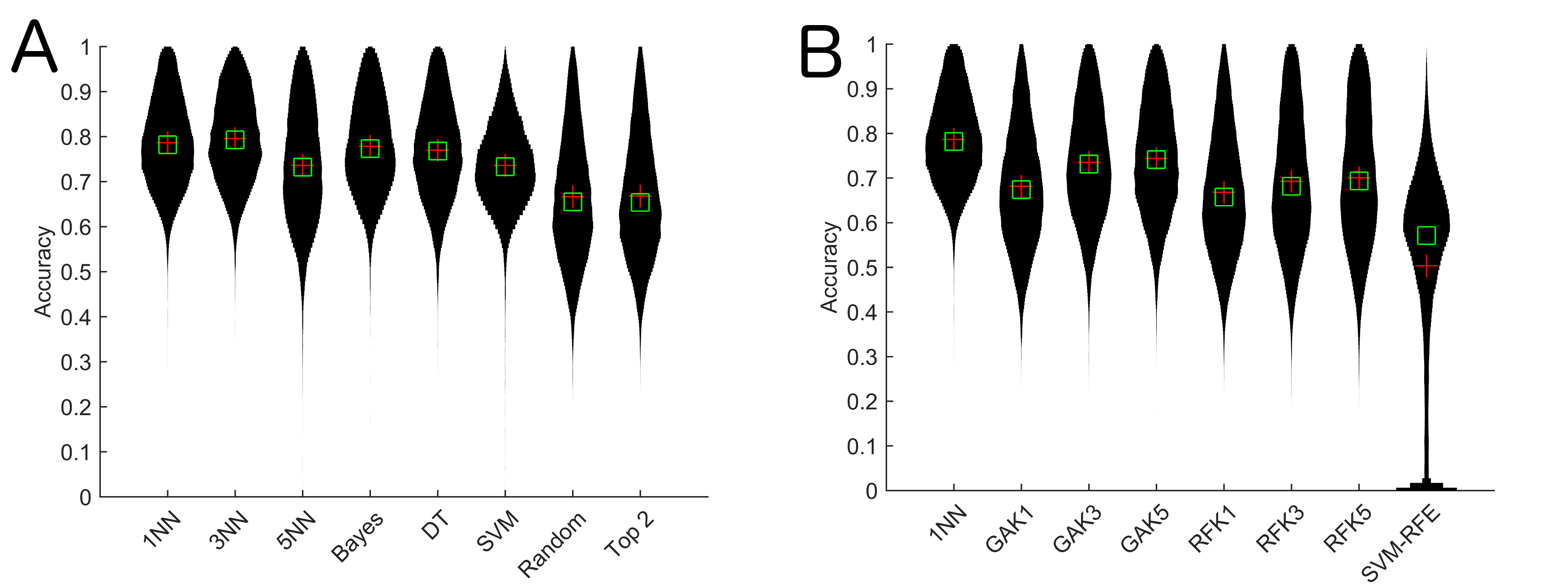

Supplement: S2 Fig — (TIF) [file pone.0148977.s002.tif]

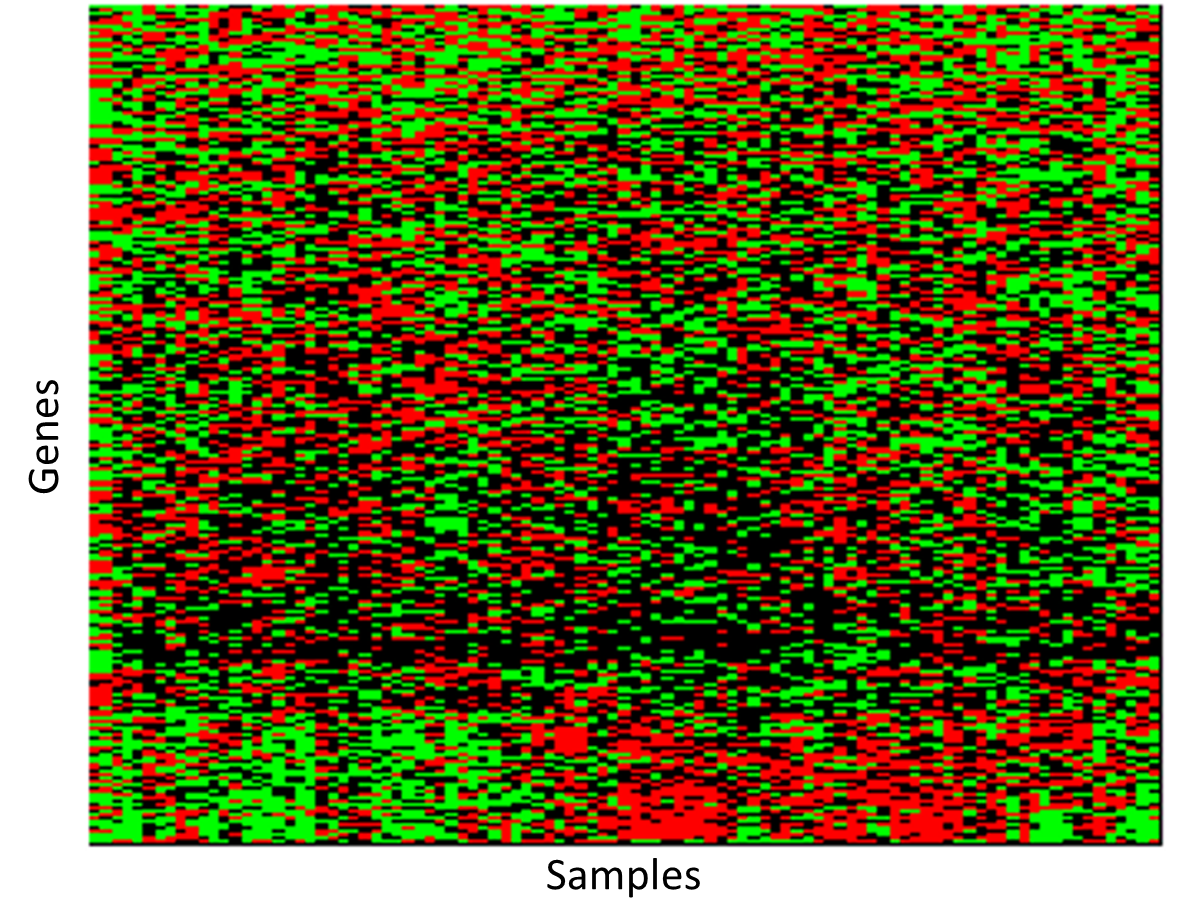

Supplement: S3 Fig — Red: Up-expressed, Green: Down-expressed, Black: Baseline. (TIF) [file pone.0148977.s003.tif]
