# Supplementary material for: A Feature Selection Algorithm to Compute Gene Centric Methylation from Probe Level Methylation Data
Source: PLoS One. 2016 Feb 12;11(2):e0148977. doi: 10.1371/journal.pone.0148977 (PMC4752315; doi:10.1371/journal.pone.0148977)
Supplement: S1 Table — (DOCX) [file pone.0148977.s005.docx]

| Cluster Number | Size | Enrichment | Most significant terms (p-val) | Other representative terms (p-val) and notes |
| --- | --- | --- | --- | --- |
| 1 | 40 | 4.39 | Atp-binding (4.4E-45), Nucleotide-binding (4.6E-38), adenyl ribonucleotide binding (1.7E-37) | Helicase (4E-12), kinase (5.8E-6), protein kinase activity (3.7E-4) |
| 2 | 4 | 3.67 | Repeat:ANK 1 (1.7E-6), Repeat:ANK 2 (1.8E-6), Ankyrin (2.9E-6) | Genes coding for ankyrin proteins |
| 3 | 45 | 3.46 | Kinase (1.8E-56), Protein Kinase – ATP binding site (2.0E-56), domain: protein kinase (2.1E-53) | Phosphorylation (1.7E-51), transferase (1.1E-47), nucleotide binding (2.1E-34) |
| 4 | 13 | 3.42 | Microtubule cytoskeleton (9.6E-15), cytoskeleton (9.1E-14), cytoskeletal part (4.1E-12) | Centrosome (2.3E-8), genes involved in regulation of cell motility |
| 5 | 5 | 3.18 | Nucleolus (8.8E-6), nuclear lumen (1.6E-4), intracellular organelle lumen (3.7E-4) | Membrane enclosed lumen (4.4E-4) |
| 6 | 6 | 3.05 | Regulation of actin filament polymerization (8.4E-13), regulation of actin filament polymerization or depolymerization (1.6E-12), regulation of actin filament length (1.9E-12) | Regulation of protein complex assembly (1.2E-11), negative regulation of actin filament depolymerization (6.3E-11) |
| 7 | 4 | 2.91 | binding site:S-adenosyl-L-methionine (1.8E-8), s-adenosyl-l-methionine (1.5E-7), methyltransferase (4.3E-7) | Genes coding for methyltransferases |
| 8 | 5 | 2.83 | Microfilament motor activity (22.0E-12), actin filament-based movement (6.3E-12), domain:Myosin head-like (9.4E-12) | Genes coding for myosin proteins |
| 9 | 6 | 2.66 | Anti-apoptosis (7.8E-12), negative reglation of apoptosis (1.2E-8), negative regulation of programmed cell death (1.3E-8) | Genes predominately related to BCL2 (BAG3, BAG4, BCL2A1, BL210). Also includes MCL1 and TNFRSF10D |
| 10 | 16 | 2.54 | Nucleotide phosphate-binding region:GTP (4.7E-28), gtp-binding (2.3E-27), Ras (2.7E-16) | Genes predominately related to the RAS oncogene family |
| 11 | 13 | 2.48 | Mitosis (2.5-22), nuclear division (2.5E-22), M phase of mitotic cell cycle (3.2E-22) | Organelle fission (4.1E-22), cell division (1.3E-17) |
| 12 | 8 | 2.38 | Guanine-nucleotide dissociation stimulator, CDC4, conserved site (1E-14), guanyl-nucleotide exchange factor activity (1.6E-14), Dbl homology (DH) domain (2.8) | Regulation of Ras protein signal transduction (2.0E-13), regulation of small GTPase mediated signal transfuction (7.2E-13), regulation of Rho protein signal transduction (9.2E-12) |
| 13 | 59 | 2.29 | Transcription regulator activity (2.7E-50), transcription regulation (2.2E-47), regulation of transcription, DNA dependent (2.2E-47) | Sequence specific DNA-binding (3.1E-29), repressor (6.0E-22) |
| 14 | 8 | 2.26 | LIM domain (6.9E-18), Zinc finger, LIM-type (2.3E-17), zinc (2.5E-7) | Metal-binding (2.1E-6) |
| 15 | 5 | 2.23 | ABC transporter-like (9E-8), ABC transporter, conserved site (1.5E-7), ATPase activity (4.2E-7) | Members of ATP-binding cassette sub-family (ABC) |
| 16 | 4 | 1.85 | Negative regulation of translation (1.5E-8), translation regulation (4.0E-8), mRNA 5’-UTR binding (2.7E-7) | Insulin-like growth factor 2 (IGF2) mRNA binding proteins |
| 17 | 5 | 1.84 | Protein tyrosine phosphatase activity (3.9E-9), tyrosine-specific phosphatase (1.5E-8), dephosphorylation (1.6E-8) | Phosphatases |
| 18 | 7 | 1.73 | Purine ribonucleoside triphosphate biosynthetic process (1.1E-10), purine nucleoside triphosphate biosynthetic process (1.1E-10) ribonucleotide triphosphate biosynthetic process (1.1E-10), | Various ATPase coding genes |
| 19 | 10 | 1.72 | Ribosomal protein (6.7E-19), structural constituent of ribosome (8.2E-18), cytostolic ribosome (1.6E-17) | Genes coding for ribosomal proteins |
| 20 | 14 | 1.62 | Wd repeat (8.2E-25), WD40 repeat (3.3E-24), WD40 repeat, conserved site | Genes with WD domain. |
