# Supplementary material for: A Feature Selection Algorithm to Compute Gene Centric Methylation from Probe Level Methylation Data
Source: PLoS One. 2016 Feb 12;11(2):e0148977. doi: 10.1371/journal.pone.0148977 (PMC4752315; doi:10.1371/journal.pone.0148977)
Supplement: S2 Table — (DOCX) [file pone.0148977.s006.docx]

| Cluster Number | Number of genes | Enrichment | Most significant terms (p-val) | Other representative terms (p-val) and notes |
| --- | --- | --- | --- | --- |
| 1 | 48 | 2.84 | Atp-binding (1.1E-51), Nucleotide-binding (6.5E-47), adenyl ribonucleotide binding (4.2E-45) | phosphorylation (4.8E-33), kinase (7.6E-40), transferase(1.9E-29) |
| 2 | 12 | 2.36 | Nucleolus (1.2E-14), nuclear lumen (3.9E-11), intracellular organelle lumen (3.7E-10) |  |
| 3 | 11 | 2.06 | Transcription regulation (1.6E-10), transcription(2.1E-10), regulation of transcription (6.8E-8) |  |
| 4 | 9 | 1.83 | Ribosomal protein (7.2E-17), ribonucleoprotein (1.8E-15), ribosome (5.6E-15) | RNA binding (2.8E-4) |
| 5 | 8 | 1.64 | Cytoskeleton (1.7E-7), microtubule cytoskeleton (2.8E-6), intracellular non-membrane-bounded organelle (1.4E-5) |  |
| 6 | 9 | 1.62 | GTP-binding (6.7E-15), guanyl nucleotide binding (5.2E-13), small GTPase mediated signal transduction (2.8E-12) | Ras oncogene related genes (RHOF, RAB3B, RAB3D, NKIRAS2, ERAS) |
| 7 | 7 | 1.47 | RNA-recognition motif, RNP-1 (3,8E-12), nucleotide-binding, alpha-beta plait (4.1E-12), RNA binding (4.8E-10) | RNA binding proteins and ribonucleoproteins |
| 8 | 6 | 1.39 | Negative regulation of ubiquitin-protein ligase activity during mitotic cell cycles (2.2E-12), negative regulation of ubiquitin-ligaase activity (2.6E-12) | Genes coding for proteasomes and ubiquitin |
| 9 | 66 | 1.38 | Regulation of transcription (1.1E-34), transcription (2.4E-24), transcription regulation (5.0E-32) |  |
| 10 | 6 | 1.28 | Homeobox (30E-10), Homebox, conserved site (5.0E-10), homeodomain-related (5.7E-10) | Homeobox proteins |
| 11 | 7 | 1.28 | Tpr-repeat (3.0E-13), tetratricopeptide-like helical (6.4E-13), tetratricopeptide region (3.9E-8) |  |
| 12 | 4 | 1.17 | SNF2-reated (6.4E-9), domain:Helicase C-terminal (1.6E-7), domain:Helicase ATP-binding (1.9E-7) | Chromodomain helicase DNA binding protein family |
| 13 | 4 | 1.09 | Protein import into nucleus, docking (1.6E-19), nuclear pore (2.3E-7_ nuclear import (2.7E-7) | Exportin 1, nucleoporin, transportin 2, importin 5 |
