# Supplementary material for: A Feature Selection Algorithm to Compute Gene Centric Methylation from Probe Level Methylation Data
Source: PLoS One. 2016 Feb 12;11(2):e0148977. doi: 10.1371/journal.pone.0148977 (PMC4752315; doi:10.1371/journal.pone.0148977)
Supplement: S3 Table — (DOCX) [file pone.0148977.s007.docx]

| Cluster number | Number of genes | Enrichment | Top terms (pval) | Other representative terms and notes |
| --- | --- | --- | --- | --- |
| 1 | 5 | 4.73 | Nucleolus (8.8E-6), nuclear lumen (1.6E-4), intracellular organelle lumen (3.7E-4) | Transcription, DNA-dependent (4.3E-2) |
| 2 | 24 | 4.08 | RNA splicing (1.0E-29), RNA processing (8.0E-29), mRNA processing (1.1E-28) | Spliceosome (6.8E-23), rna-binding (2.3E-10) |
| 3 | 13 | 2.48 | Cytoskeleton (1.5E-18), cytoplasm (7.2E-10), microtubule cytoskeleton (4.7E-9) |  |
| 4 | 11 | 2.25 | Ribosomal protein (6.3E-21), ribonucleoprotein (3.5E-19), ribosome (1.5E-18) | Group of genes coding for mitochondrial ribosomal proteins |
| 5 | 134 | 2.2 | Transcription regulation (1.9E-45), zinc (4.1E-45), transcription (1.3E-43) | Transcription regulation |
| 6 | 13 | 2.03 | Ubl conjugation pathway (1E-19), modification-dependent protein catabolic process (3E-17), modification-dependent macromolecule catabolic process (3E-17) | Ubiquitin proteins, proteolysis (4.7E-14) |
| 7 | 5 | 1.84 | Repeat: ANK1 (2.1E-8), repeat ANK2 (2.1E-8), ank repeat(2.4E-8 | Ankyrin proteins |
| 8 | 9 | 1.68 | Mitosis (5.8E-17), cell division (1.1E-15), nuclear division (4.3E-15) |  |
| 9 | 9 | 1.4 | Repeat:WD3 (1.2E-15), repeat:WD 2 (1.6E-15), repeat: WD1 (1.6E-15) | WD containing proteins |
| 10 | 6 | 1.17 | Kelch repeat (1.7E-10), repeat:Kelch 4 (8.2E-10), repeat:Kelch 1 (8.7E-10) |  |
| 11 | 4 | 1.13 | Aminoacyl-tRNA synthetase (7.7E-9), tRNA aminoacylation (3.7E-8), amino acid activation (3.7E-8) | tRNA synthetases |
| 12 | 4 | 1.09 | Protein tyrosine phosphatase (1.1E-7), protein tyrosine phosphatase, active site (1.5E-7), protein tyrosine phosphatase activity (5E-7) | Protein tyropsine phosphatases |
| 13 | 19 | 1.02 | Transport (7.2E-14), mitochondrial envelope (4.3E-13), mitochondrion (5.8E-13) |  |
