# Supplementary material for: A Feature Selection Algorithm to Compute Gene Centric Methylation from Probe Level Methylation Data
Source: PLoS One. 2016 Feb 12;11(2):e0148977. doi: 10.1371/journal.pone.0148977 (PMC4752315; doi:10.1371/journal.pone.0148977)
Supplement: S4 Table — (DOCX) [file pone.0148977.s008.docx]

| Cluster Number | Number of genes | Enrichment | Most significant terms (p-val) | Other representative terms (p-val) and notes |
| --- | --- | --- | --- | --- |
| 1 | 4 | 3.3 | GTPase activation (5.5E-7), domain:PH (1.9E-6), Pleckstrin homology (4.5E-6) | Rho GTPases |
| 2 | 5 | 2.5 | Atp-binding (2.2E-5), nucleotide-binding(5.9E-5), adenyl ribonucleotide binding (1.8E-4) |  |
| 3 | 17 | 2.14 | Protein kinase – core (8.7E-23), kinase (2.7E-21), protein kinase – atp binding site (1.2E-20) | Phosphorylation (1.9E-20), nucleotide-binding (1.9E-15), transferase (7.3E-16) |
| 4 | 5 | 1.86 | Zinc (1.7E-4), metal-binding (5.7E-4), zinc ion binding (1E-3) |  |
| 5 | 5 | 1.59 | GTP-binding (8.4E-8), guanyl nucleotide binding (7.4E -7), guanyl ribonucleotide binding (4.7E-7) |  |
| 6 | 4 | 1.59 | Guanine nucleotide dissociation stimulator, CDC24, conserved site (5.2E-8), Dbl homology (DH) domain (6.8E-8), Rho guanyl nucleotide exchange factor activity (1.8E-7) | Regulation of apoptosis (2.1E-4) |
| 7 | 4 | 1.41 | EGF-like, type 3 (1.6E-6), egf-like domain (1.7E-6), EGF-like (1.7E-6) |  |
| 8 | 5 | 1.26 | DNA/RNA helicase (4.7E-8), domain:Helicase C-terminal (6.4E-7), Helicase:ATP-binding (7.4E-7) | Chromodomain helicases |
| 9 | 8 | 1.18 | Repeat:WD 3 (9.1E-14), repeat:WD2 (1.2E-13), repeat:WD1 (1.2E-13) | WD containing proteins |
| 10 | 4 | 1.17 | Nucleoplasm (3.3E-4), transcription regulation (1.2E-3), transcription (1.2E-3) |  |
